# Lysine-mediated hydroxyethyl starch-10-hydroxy camptothecin micelles for the treatment of liver cancer

**DOI:** 10.1080/10717544.2020.1745329

**Published:** 2020-03-31

**Authors:** Guofei Li, Mingming Zhao, Limei Zhao

**Affiliations:** Department of Pharmacy, Shengjing Hospital, China Medical University, Shenyang, China

**Keywords:** Hydroxyethyl starch conjugate, chemotherapy, liver cancer therapy, targeted drug delivery, amino acid transporters

## Abstract

Liver cancer is a malignant tumor with extremely high morbidity and mortality. At present, traditional chemotherapy is still the most commonly used therapeutic approach. However, serious side effects lead to the treatment of liver cancer is not ideal. Therefore, it is imperative to develop a new drug delivery system based on nanotechnology and liver cancer microenvironment. In this study, a pH/reduction/α-amylase multi-sensitive hydroxyethyl starch-10-hydroxy camptothecin micelles (HES-10-HCPT-SS-Ly) targeting over-expressed amino acid (AA) transporters on the surface of liver cancer cell by applying lysine were successfully synthesized. The prepared micelles showed regular structure, suitable particle size, and intelligent drug release property. Compared with conventional HES-10-HCPT micelles and 10-HCPT injection, HES-10-HCPT-SS-Ly micelles demonstrated better *in vitro* anti-proliferative capability toward human liver cancer Hep-G2 cells and greater antitumor efficiency against nude mouse with Hep-G2 tumor. These findings suggest that HES-10-HCPT-SS-Ly micelles may be a promising nanomedicine for treatment of liver cancer.

## 1. Introduction

10-Hydroxy camptothecin (10-HCPT) is a very common chemotherapy drug, which has strong antitumor activity against multiple types of tumors via inhibiting the DNA topoisomerase I (Li et al., 2016; Tang et al., 2017; Xing et al., 2019). Therefore, 10-HCPT is a classic chemotherapy drug commonly used in clinical practice. However, with the emergence of new types of chemotherapy drugs and continuous innovation of treatment methods, the therapeutic status of 10-HCPT has been greatly challenged because of its defects, such as poor water solubility and stability, short half-life, serious side reaction, low efficacy, etc. (Liu et al., 2017). Therefore, how to improve the defects of 10-HCPT to increase its clinical efficacy is an urgent problem to be solved.

Recently, the construction of drug delivery systems based on nanotechnology to load and deliver chemotherapy drugs is the most commonly used technique. Among a variety of approaches, polymer–drug conjugated micelles are a well known and widely exploited technique because it can improve the defect of the properties of the coupled drug by virtue of the properties of the polymer used (Sarika et al., 2015; Almawash et al., 2018; Zou et al., 2018; Lubtow et al., 2019; Shi et al., 2019). For example, Lv prepared a bottlebrush conjugate by attaching 10-HCPT and methoxypolyethylene glycols (mPEG) into the polyester backbone (Lv et al., 2019). The synthesized bottlebrush mPEG-HCPT conjugate showed high drug loading, good stimuli-responsive, effective internalization into tumor cells and excellent anticancer efficacy. Zhou synthesized a matrix metalloproteinases (MMPs)-sensitive N-(2-hydroxypropyl) methylacrylamide polymer–doxorubicin conjugate to prolong the circulation time and enhance its anti-tumor efficacy (Zhou et al., 2019). The results showed that the circulation time (about 20.1 h) and tumor growth inhibition (around 70%) of the conjugate improved significantly in comparison with free doxorubicin. In addition, the polymer–drug conjugated micelles have other advantages compared with the traditional micelles, including diversification of drug-loaded forms, stimuli responsiveness of coupled bonds, and the ability to simultaneously load multiple drugs or targets. Therefore, polymer–drug conjugated micelles may be a powerful weapon to improve 10-HCPT deficiency and deliver it to tumor tissue.

Nanomedicine can passively target tumor tissues by means of the EPR effect, but how to enter the cell is the key to improving the efficacy for drugs that targeted the nucleus (Maeda, 2017). Active targeting technology is the most commonly used method to increase the uptake of drugs by cells by utilizing the specific microenvironmental differences of tumor cell surface, including over-expressed antigens, receptors, enzymes, transporters, etc. (Li and Zhao, 2019; Mohammed et al., 2019; Zhang et al., 2019). It is well known that the progression of the tumor requires a large amount of nutrients, including amino acids (AA), glucose, etc., which leads to overexpression of AA transporters and glucose transporters on the surface of tumor cells (Madunic et al., 2018; Hafliger and Charles, 2019; Ogawa et al., 2019; Sztandera et al., 2019). Moreover, transporter-mediated drug transport exhibited higher specificity and efficiency than receptor-ligand-mediated active targeting in terms of transport efficiency. Therefore, transporter-mediated drug transport has become a research hotspot for drug delivery. However, although active targeting can promote nanomedicine into cells, the uptake efficiency of drugs is often undesirable due to large molecular weight (Mw) and complex spatial structure of polymer. Therefore, the delivery of small molecule drugs through active targeting may be an more effective means of increasing cellular uptake.

The choice of polymer is essential for the preparation of polymer–drug conjugated micelles. In this study, hydroxyethyl starch (HES) was used as polymer carrier to link 10-HCPT because HES possesses excellent physical and chemical properties (Hu et al., 2016; Paleos et al., 2017; Tatara, 2018), which make HES become an important biomedical material widely used in the clinical and pharmaceutical excipients, including plasma substitute, freeze-dried protective agent, and polymer carrier.

In this study, lysine modified HES-10-HCPT micelles (HES-10-HCPT-SS-Ly) were prepared to transport 10-HCPT, and its *in vitro* and *in vivo* behaviors were also evaluated systematically, including drug release, cytotoxicity, pharmacokinetics, pharmacodynamics, and tissue distribution. The results showed that HES-10-HCPT-SS-Ly micelles can significantly improve the physical and chemical properties of 10-HCPT. More importantly, HES-10-HCPT-SS-Ly micelles exhibited better long circulating effect in plasma and cytotoxicity compared with 10-HCPT injection. Meanwhile, the prepared micelles showed satisfactory anti-tumor effect due to the existence of active targeting between Ly and AA transporters.

## 2. Materials and methods

### 2.1. Materials and reagents

10-Hydroxy camptothecin (99%) was purchased from Wuhan Lishizhen Pharmaceutical Co., Ltd. (Wuhan, China). 10-Hydroxy camptothecin injection (1 mg/mL) was purchased from Shengjing Hospital of China Medical University (Shenyang, China). Hydroxyethyl starch (HES, 130 kDa/0.4) was obtained from Chongqing Daxin Pharmaceutical Co., Ltd. (Chongqing, China). The Mw of HES is 130 kDa and the degree of substitution is 0.4. 2,2′-dithiodipyridine (99%), dithiothreitol (98%), 3-mercaptopropionic acid (98%), N,N′-dicyclohexycarbodiimide (99%), 4-dimethylaminopyridine (99%) and N-hydroxysuccinimide (99%), N-(tert-butoxycarbonyl)glycine (98%), succinic anhydride (99%), di-tert-butyl dicarbonate (99%) were purchased from Shanghai Darui Fine Chemical Co., Ltd. (Shanghai, China). 3-(4,5-Dimethyl-thiazol-2-yl)-2,5-diphenyl tetrazolium bromide and human liver cancer Hep-G2 cell line were purchased from Shanghai Institutes for Biological Sciences (Shanghai, China).

### 2.2. Animals

Twelve male Wistar rats weighting 180 ± 20 g and 20 nude mice weighing 20–25 g were obtained from Beijing HFK Bioscience Co. Ltd. (Beijing, China). All animal-use procedures complies with relevant regulations. Rats were housed in accordance with the following conditions: the interval of illumination is 12 hours, the indoor temperature is basically constant, about 25 °C, the relative humidity was 45.0–60.0%. The rats were fasted for 12.0 h but had free access to water prior to administration.

### 2.3. Synthesis of HES-10-HCPT-SS-Ly conjugates

#### 2.3.1. Synthesis of 3-(2-pyridyldithio) propionic acid (PDP)

3-(2-Pyridyldithio) propionic acid was prepared based on a method reported by Li et al. (2017). 2,2′-Dithiodipyridine (4.5 g) and 2.5 mL glacial acetic acid were dissolved in 70 mL ethanol. Then, 3-mercaptopropionic acid (1.0 g) dissolved in 25 mL ethanol was added dropwise to the above solution under continuous stir. The mixture was stirred overnight at room temperature. After the reaction was completed, the solvent was removed by vacuum evaporation.

#### 2.3.2. Synthesis of 10-HCPT-PDP

10-HCPT-PDP was synthesized via an esterification reaction between PDP and 10-HCPT. 10-HCPT (0.37 g), PDP (0.30 g), and DCC (1.20 g) were dissolved in 50 mL dichloromethane (DCM) and stirred at room temperature for 24 h. Then, the by-products (N,N′-dicyclohexylurea) of the reaction are removed by the filtration, and the corresponding filtrate was processed by using rotary evaporation.

#### 2.3.3. Synthesis of 10-HCPT-MPA

10-HCPT-PDP (0.28 g) and DTT (0.70 g) were simultaneously dissolved in 50 mL DCM and stirred continuously for 12 h. After extracted with ultra-pure water for three times, the organic phase was processed under vacuum.

#### 2.3.4. Synthesis of Ly-PDP

Ly-PDP was synthesized via an esterification reaction between PDP and Boc-Lys-OtBu. Boc-Lys-OtBu (0.15 g), PDP (0.06 g), and DCC (0.1 g) were dissolved in 50 mL DCM and stirred for 24 h. Then, 3 mL of trifluoroacetic acid was added to the solution and stirred for 3 h. The by-products (N,N′-dicyclohexylurea) of the reaction are removed by the filtration and the corresponding filtrate was processed by using rotary evaporation.

#### 2.3.5. Synthesis of 10-HCPT-SS-Ly

10-HCPT-SS-Ly was synthesized by a thiol-disulfide exchange reaction between 10-HCPT-MPA and Ly-PDP. Ly-PDP (200.0 mg) and 10-HCPT-MPA (100.0 mg) were dissolved in 15 mL DCM and stirred for 24 h. After extracted with ultra-pure water for three times, the organic phase was processed under vacuum.

#### 2.3.6. Synthesis of HES-CHO

HES-CHO was synthesized according to the corresponding reference reported by Sagnella et al. (2014). Briefly, 4.3 g sodium periodate and 3.0 g HES were simultaneously dissolved in 100 mL deionized water and stirred overnight in the dark. When the reaction is over, the products were processed using cellulose membrane (3500 g/mol) and lyophilized to obtain final HES-CHO.

#### 2.3.7. Synthesis of HES-10-HCPT-SS-Ly

HES-CHO (1.6 g) and 10-HCPT-SS-Ly (0.2 g) were dissolved in 20 mL DMSO and stirred at for 48 h. When the reaction is over, the mixture was dialyzed against 20% DMSO-deionized water solution to remove reactant. Then, the precipitates of HES-10-HCPT-SS-Ly can be obtained by adding the solutions to ice ethanol. The above precipitates were processed under vacuum, and final HES-10-HCPT-SS-Ly conjugates were obtained.

### 2.4. Preparation of HES-10-HCPT-SS-Ly micelles

HES-10-HCPT-SS-Ly conjugates can self-assemble into micelles by virtue of its amphiphilicity. In this study, dialysis is used to prepare HES-10-HCPT-SS-Ly micelles. Briefly, HES-10-HCPT-SS-Ly conjugates were dissolved with 10 mL DMSO solution and laced in a dialysis bag and the Mw cutoff of dialysis bag was 3500–5000 g/mol. The dialysis bag containing HES-10-HCPT-SS-Ly conjugates solution was dialyzed for 24 h. Finally, the above solutions were freeze-dried to obtain the HES-10-HCPT-SS-Ly micelles. The properties of the HES-10-HCPT-SS-Ly micelles, including morphology, particle size, potential and dispersion state were characterized by corresponding technical means.

### 2.5. *In vitro* release of 10-HCPT

The *in vitro* release behavior of 10-HCPT from micelles was studied in 10 mM dithiothreitol solutions, phosphate buffer solutions (PBS, pH 5.5, 6.8, 7.4) containing 10 mM dithiothreitol, PBS (pH 5.5, 6.8, and 7.4) containing amylase and 10 mM dithiothreitol respectively using the dialysis method. Briefly, the HES-10-HCPT-SS-Ly micelles were placed in a dialysis bag and diluted with the above release medium to a concentration of 0.5 mg/mL, respectively. The volume of dialysis fluid is 5.0 mL and the Mw cutoff was 3500 g/mol. 0.2 mL of dialysis fluid would be taken out at a predetermined time and the concentration of 10-HCPT by using high performance liquid chromatography was determined.

### 2.6. Cytotoxicity assay and uptake test

The *in vitro* cytotoxicity of HES-10-HCPT-SS-Ly micelles was evaluated by MTT assay and Hep-G2 cell lines were used as model object. The Hep-G2 cells were seeded by using 96-well plates and the cell density was 6 × 10^3^. The temperature of cell culture was 37 °C and the concentration of CO_2_ was 5%. The culture medium was removed after 24 h and the micelle solutions with different concentrations are added. The incubation time was 48 h and 72 h respectively to fully evaluate the relationship between cytotoxicity and culture time. Then, 50 μL MTT (0.25 mg/mL) was added to each well and the supernatant was removed. The precipitated formazan crystals were dissolved by DMSO solution after 4 h. The OD values were determined by using an ELIASA reader and the detection wavelength was 492 nm. In addition, cellular uptake of HES-10-HCPT-SS-Ly micelles was also assessed in Hep-G2 cell line. Similarly, the culture medium was removed and different 10-HCPT preparations were added with concentrations of 1.0 mg/mL 24 h after incubation. The 96-well plate was incubated at room temperature on a plate shaker for 24 h, the cells were then washed three times with PBS solution. Briefly, cells were collected and lysed using a French Pressure Cell, the lysate was clarified by centrifugation, and the supernatant liquid was processed by applying liquid–liquid extraction. Then, the concentration of 10-HCPT in Hep-G2 cells was determined by HPLC–MS/MS method.

### 2.7. Pharmacokinetics of the prepared micelles

The pharmacokinetics of HES-10-HCPT-SS-Ly micelles and 10-HCPT injection in Sprague-Dawley rats were evaluated in this study (*n* = 6). Briefly, HES-10-HCPT-SS-Ly micelles and 10-HCPT injection were injected, respectively. The dose of 10-HCPT was 1.5 mg/kg in both groups. 0.2 mL blood was collected 5 min, 10 min, 15 min, 30 min, 60 min, 2 h, 4 h, 6 h, 8 h, 12 h, 24 h, 48 h, and 72 h after administration. The concentration of 10-HCPT in plasma was determined by HPLC–MS/MS method.

### 2.8. *In vivo* pharmacodynamics evaluations

Next, the antitumor effect of HES-10-HCPT-SS-Ly micelles was evaluated in nude mice bearing Hep-G2 tumor xenograft. The mice were treated with HES-10-HCPT micelles solution, HES-10-HCPT-SS-Ly micelles solution, 10-HCPT injections, and normal saline. The dose of 10-HCPT was 1.5 mg/kg. Mice were given corresponding preparations on Mondays and Thursdays for four weeks. The tumors were totally excised and precisely weighed after the end of the dosing period. Tumor inhibition rate (%)=(*V*_blank_ – *V*_sample_)/*V*_blank_×100%.

### 2.9. Tissue distribution test of HES-10-HCPT-SS-Ly micelles

Tissue distribution test of HES-10-HCPT-SS-Ly micelles was evaluated by using Sprague-Dawley rats. 10-HCPT injection was used as reference preparation.

HES-10-HCPT-SS-Ly micelles and 10-HCPT injection were administrated via tail vein at a dose of 1.50 mg/kg. The mice were killed at 0.5, 1, 4, 12, 24, and 48 h respectively after drug administration. Liver, heart, lung, spleen, kidney, marrow, and tumor of mice were collected. Then, each organization will be processed according to standard operating procedures. Briefly, each tissue would be cut into pieces, weighed and homogenized with methanol. Four hundred milliliters of tissue homogenate and 100 mL diphenhydramine hydrochloride (50 ng/mL) of the IS solution were added in a 2.0 mL EP tube, and vortexed for 1 min. The 10-HCPT present in each tissue was extracted by mixed solution of ethylacetate (95) and isopropanol (5) and analyzed by HPLC–MS/MS system.

## 3. Results and discussion

### 3.1. Synthesis and characterization of HES-10-HCPT-SS-Ly micelles

**Scheme 1.**
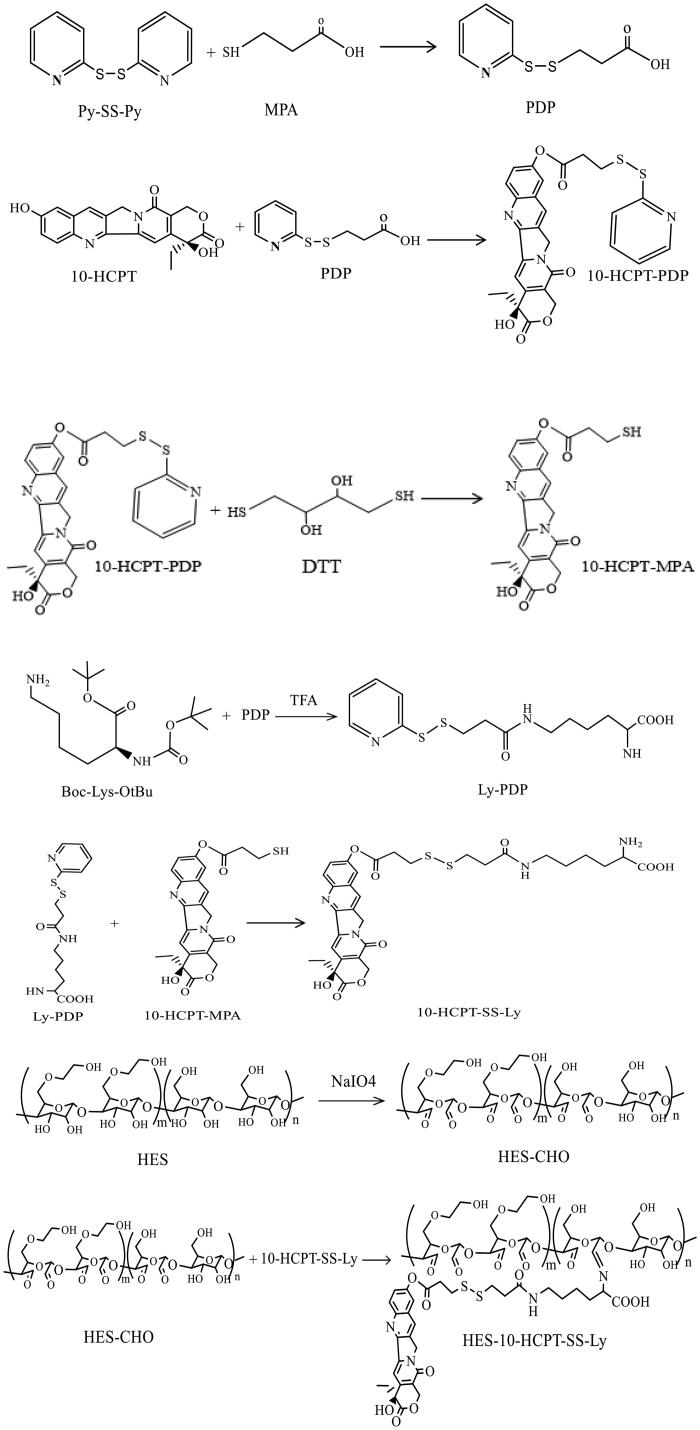
Synthetic route of HES-10-HCPT-SS-Ly conjugates.

**Figure 1.**
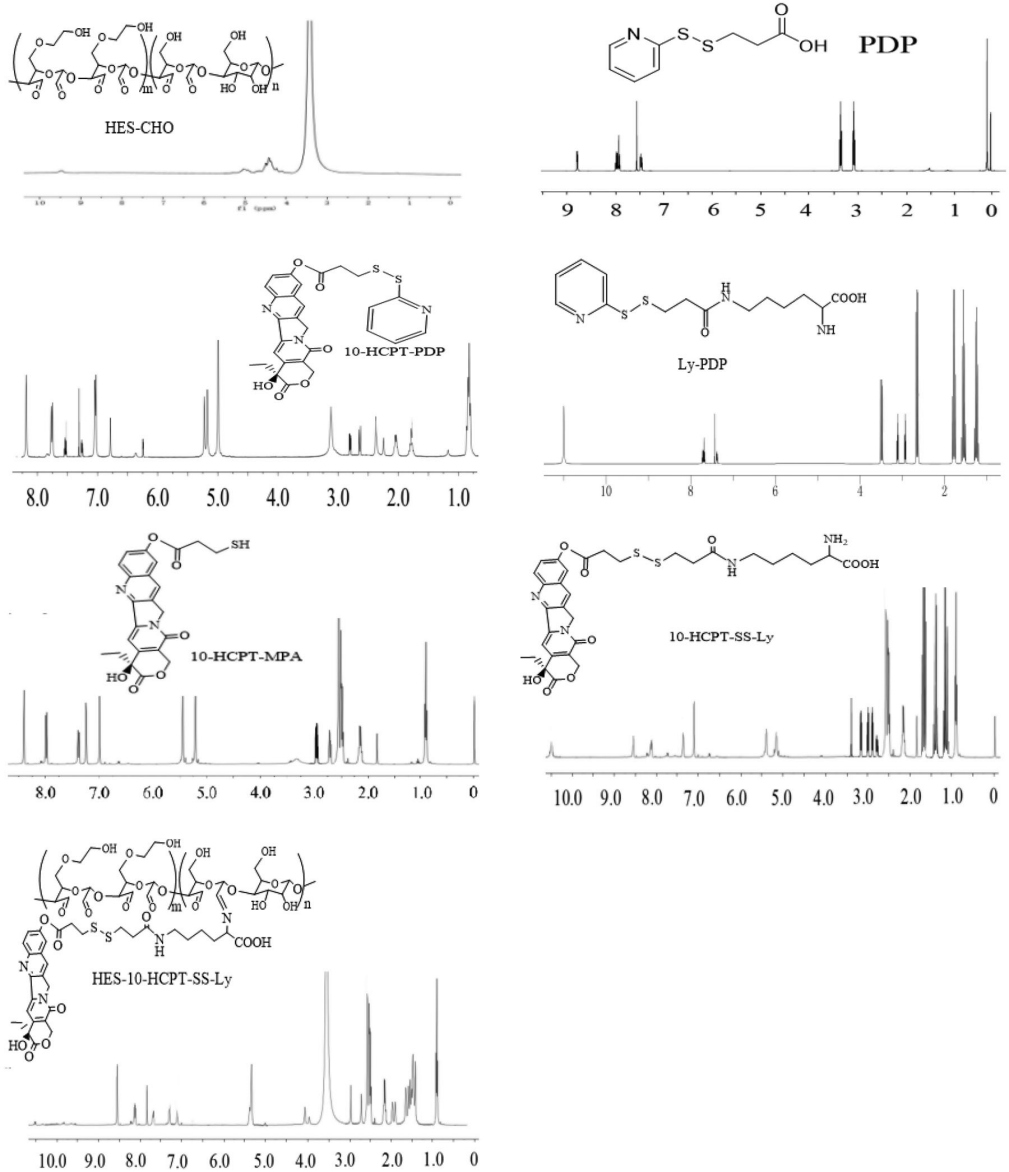
^1^H NMR spectrums of HES-CHO, PDP, 10-HCPT-PDP, Ly-PDP, 10-HCPT-MPA, 10-HCPT-SS-Ly, and HES-10-HCPT-SS-Ly.

In this paper, HES (130 kDa/0.4) with average Mw of 130 kDa and molar substitution of hydroxyethyl 0.4, was used to prepare HES-10-HCPT-SS-Ly conjugates. The specific synthesis process of HES-10-HCPT-SS-Ly conjugates is shown in Scheme 1. ^1^H NMR was used to confirm the structure of the products, and the spectrum is exhibited in Figure 1. HES-CHO: NMR peak of aldehyde groups appeared at 8.1 ppm with the opening of the glucose ring and the aldehyde group of HES. Meanwhile, the NMR peaks at chemical shift 5.6–4.0 ppm should be H on the hydroxyl group of the sugar ring and the C1. PDP: the NMR peak of the protons of methylene and pyridyl can be found at 3.0–3.5 ppm and 7.5–8.0 ppm, respectively, which was consistent with the PDP signal. 10-HCPT-PDP: it is easy to obtain 10-HCPT-PDP by the reaction between the hydroxyl group of 10-HCPT and the carboxyl group of PDP. The proton signals of PDP can be seen from the NMR spectrum of 10-HCPT-PDP compared with the NMR spectra of 10-HCPT. Ly-PDP: PDP was coupled to the amino group of Boc-Lys-OtBu to produce Boc-Lys-PDP via an reaction between amino group and carboxyl group. Then, the BOC group is removed by the action of 30% trifluoroacetic acid to obtain the final product Ly-PDP. It should be a characteristic peak of methylene of Ly and the protons of methylene of PDP at 1.25–2.65 ppm and 3.0–3.5 ppm, respectively. Additionally, the characteristic peak of amino group at 11.0 ppm can be seen in the NMR spectrum. 10-HCPT-MPA: the 10-HCPT-MPA was obtained by the reduction reaction 10-HCPT-PDP. It can be seen from the NMR spectrum that the proton signals of pyridyl disappeared with the synthesis of 10-HCPT-MPA. 10-HCPT-SS-Ly: 10-HCPT was linked to Ly via a disulfide-thiol exchange reaction between 10-HCPT-MPA and Ly-PDP. The characteristic resonance signals of 10-HCPT and Ly appear at 1.0–3.0 (methylene), 5.0–5.5 (methylene on 10-HCPT ring), 7.0–7.5 (H of olefin on 10-HCPT ring), 8.0–8.6 (H of olefin on 10-HCPT ring), and 11.0 ppm (amino group of lysine). Meanwhile, the chemical shifts of the characteristic peaks are shifted to some extent because of the formation of disulfide bonds in comparison with the NMR spectrum of 10-HCPT-MPA and Ly-PDP. Finally, HES-10-HCPT-SS-Ly conjugate was synthesized via coupling 10-HCPT-SS-Ly to HES by imine bond. It can be seen that the amino resonance peak of Ly disappears with the formation of imine bond, and the resonance peak of imine bond appeared at 7.8 ppm. Meanwhile, the vibration peak of aldehyde (HES-CHO) has undergone significant changes. In addition, the inherent characteristic resonance of 10-HCPT and Ly can be found on the ^1^H NMR spectrum. Thus, the synthesis of HES-10-HCPT-SS-Ly conjugate is successful.

### 3.2. Preparation of HES-10-HCPT-SS-Ly micelles

It is well known that HES has good hydrophilicity, while 10-HCPT is a hydrophobic substance. Therefore, HES-10-HCPT-SS-Ly conjugate showed obvious amphiphilicity and can self-assembly easily into micelles. It was found that the critical micelles concentration (CMC) of HES-10-HCPT-SS-Ly micelles was 11.6 μg/mL. Meanwhile, HES-10-HCPT-SS-Ly micelles showed good morphology observed by transmission electron microscopy and suitable particle size (about 71.4 ± 9.4 nm) was measured by dynamic light scattering. Additionally, it was found that the speed of dialysis has a significant effect on the formation of micelles, which may be related to the decrease in the solubility of HES.

### 3.3. *In vitro* drug release of the micelles

**Figure 2.**
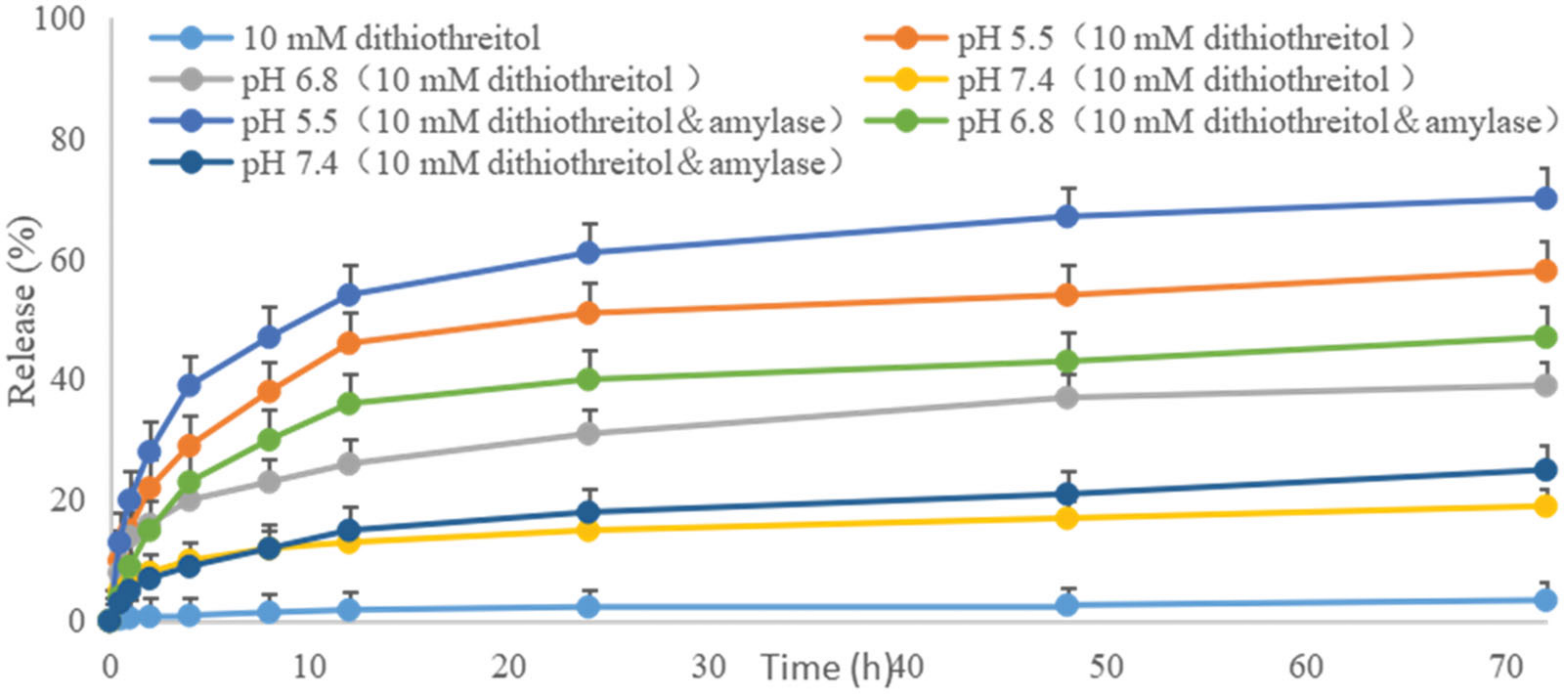
Cumulative release (%) of 10-HCPT from micelle in different release medium at 37 °C.

The release degree of 10-HCPT from micelles was studied in this study. The release percentages of the drugs versus time are plotted in Figure 2.

As depicted in Figure 2, the amount of 10-HCPT released from HES-10-HCPT-SS-Ly conjugate was 3% in 72 h at pH 7.4. It can be concluded that 10-HCPT is almost not released in the presence of only dithiothreitol. The reason may be as follows: HES-10-HCPT-SS-Ly conjugate can only be degraded into HES-10HCPT in the presence of dithiothreitol because the Schiff base bond between HES and 10-HCPT is not sensitive to dithiothreitol. HES-10-HCPT conjugate cannot pass through the dialysis bag due to the large Mw, which resulted in low release of 10-HCPT. In the PBS solution containing 10 mM dithiothreitol, the release of 10-HCPT was significantly different from that of 10 mM dithiothreitol solutions. It was found that the release of 10-HCPT has been significantly improved with the decrease of pH, reaching 19% (pH 7.4), 39% (pH 6.8), and 58% (pH 5.5) after 72 hours, respectively. It can be seen that the release of 10-HCPT was closely related to pH of release medium. This may be due to the breakage of the Schiff base bond between 10-HCPT and HES and the depolymerization of the micelles increased as the pH decreased. That is to say, the presence of Schiff base bond makes the release of 10-HCPT to have acid-sensitive property, thereby avoiding the early release of the drug. The release of 10-HCPT is further increased with the addition of α-amylase to the release medium, and reaching 25% (pH 7.4), 47% (pH 6.8), and 70% (pH 5.5) after 72 hours. The reason may be as follows: although HES has a large Mw and a complex spatial structure, it can be hydrolyzed into glucose units by α-amylase. With the disintegration of the HES structure, the space protection effect of HES on 10-HCPT is gradually weakened, resulting in a significant increase in the release of 10-HCPT under acidic conditions. Therefore, 10-HCPT can be rapidly released in tumor tissues due to the low pH environment and high concentration of α-amylase in the tumor area. Additionally, the concentration of glutathione in tumor cells is much higher than that of normal cells and tumor tissues, ensuring that 10-HCPT-Ly can rapidly release drugs after entering tumor cells.

### 3.4. *In vitro* cytotoxicity test

**Figure 3.**
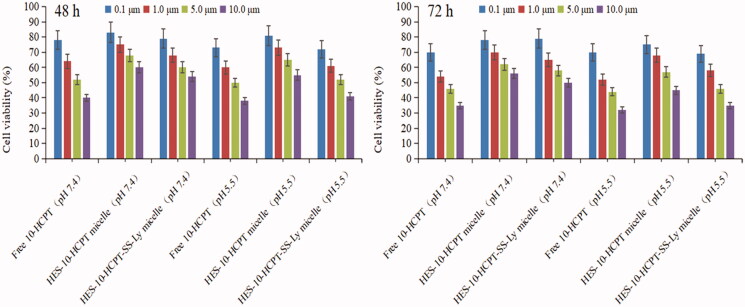
Results of MTT assay on Hep-G2 cells after incubation of 48 and 72 h with drugs solutions and micelle at various concentrations.

**Figure 4.**
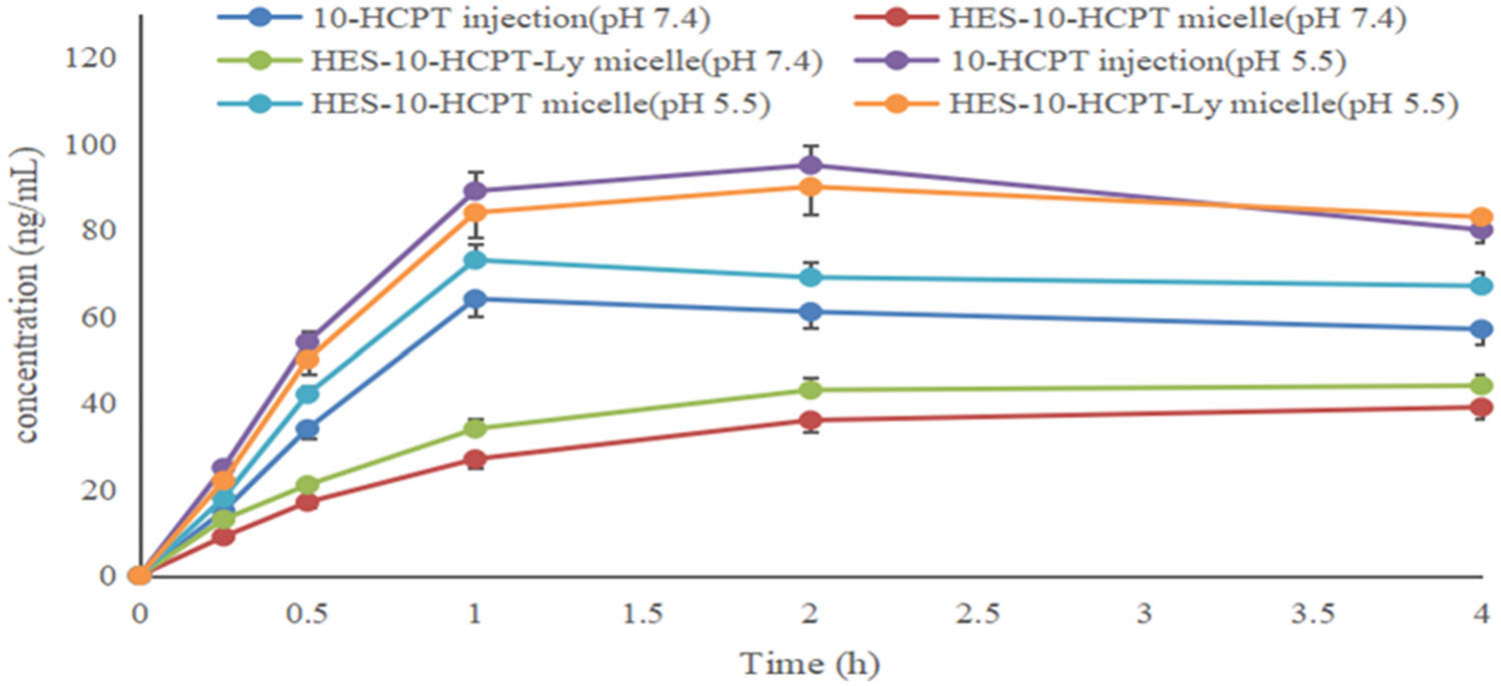
The cell drug concentration versus time curves of micelle and 10-HCPT injection.

The cytotoxicity of HES-10-HCPT-SS-Ly micelles on Hep-G2 cells and uptake test were studied in this paper and the results are shown in Figures 3 and 4.

It can be seen that no obvious inhibition on Hep-G2 cells by aldehydeated HES was found at different concentrations. The cytotoxicity of free 10-HCPT was higher than that of HES-10-HCPT micelles and HES-10-HCPT-SS-Ly micelles in a culture medium at pH 7.4, hence cell survival in the free 10-HCPT group was lower than that of the other groups, and the IC_50_ value of free 10-HCPT group, HES-10-HCPT micelles group, HES-10-HCPT-SS-Ly micelles group was 5.1 μM, 11.7 μM, and 9.9 μM, respectively. In contrast, the relative cytotoxicity of the HES-10-HCPT micelles and HES-10-HCPT-SS-Ly micelles was significantly increased at pH 5.5, especially for HES-10-HCPT-SS-Ly micelles, and the IC_50_ value of three groups was 4.3 μM, 8.4 μM, and 4.8 μM, respectively. At the same time, similar results were obtained in cellular uptake of 10-HCPT preparations by Hep G2 cells after a 72 h incubation. It was found that free 10-HCPT was more easily taken up by Hep G2 cells at pH 7.4 than HES-10-HCPT micelles and HES-10-HCPT-SS-Ly micelles. The uptake efficiency of three 10-HCPT preparations increased significantly with the decrease of medium pH value, especially for HES-10-HCPT-SS-Ly micelles. The potential reasons why the study results were so different may be as follows. (1) The small, free 10-HCPT passed through cell membranes more easily and entered the cells. In contrast, micelles cannot enter into cell quickly because of absorption mode and water-soluble surface. Thus, free 10-HCPT showed higher cytotoxicity and uptake efficiency than HES-10-HCPT micelles and HES-10-HCPT-SS-Ly micelles. (2) 10-HCPT mainly existed as carboxylate at pH 7.4, and the carboxylate form of 10-HCPT was converted into the lactone form with the reduction of pH. While carboxylate 10-HCPT had a greater polarity than the lactone form of 10-HCPT. It is well known that highly polar substances do not easily cross cell membranes. Therefore, 10-HCPT preparations exhibited higher cytotoxicity and uptake efficiency at pH 5.5 than 7.4. (3) The bonds between HES and 10-HCPT were more easily broken at pH 5.5, hence 10-HCPT can be rapidly released and taken up by Hep G2 cells. Meanwhile, Ly-mediated effects would further increase 10-HCPT uptake. Then, HES-10-HCPT-SS-Ly micelles showed greater cytotoxicity as pH decreased in comparison with HES-10-HCPT micelles. To sum up, the micelles modified with Ly can enter cells more easily through the identification between Ly and AA transporters.

### 3.5. Pharmacokinetics of HES-10-HCPT-SS-Ly micelles

**Figure 5.**
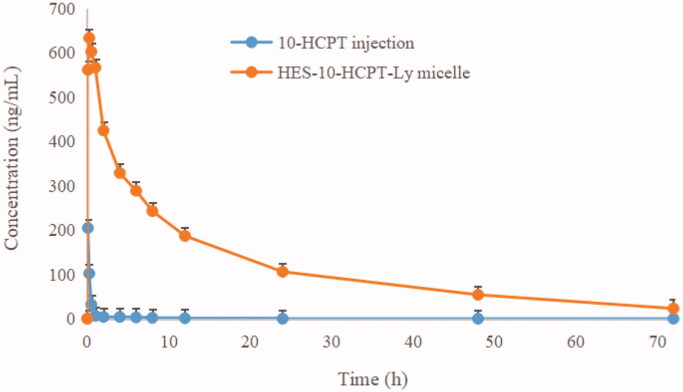
The rat plasma concentration versus time curves of micelle and 10-HCPT injection after intravenous administration.

Rat pharmacokinetics was studied to assess the long circulating effect of HES-10-HCPT-SS-Ly micelles. The results are shown in Figure 5 and Table 1.

**Table 1.** Pharmacokinetic parameters of 10-HCPT after intravenous administration of different formulations (mean ± SD).

Pharmacokinetic parameters	10-HCPT injection	HES-10-HCPT-Ly micelles
AUC_(0–_*_t_*_)_ (μg/L·h)	80.2 ± 13.8	3874.5 ± 512.5^a^
AUC_(0–∞)_ (μg/L·h)	82.5 ± 14.3	3876.6 ± 514.7^a^
CL_z_ (L/h/kg)	52.6 ± 16.7	0.112 ± 0.015^a^
*C*_max_ (μg/L)	205.4 ± 43.6	603.3 ± 84.5^a^
*t*_1/2_ (h)	0.13 ± 0.03	4.58 ± 1.42^a^
*T*_max_ (h)	0.083 ± 0.00	0.167 ± 0.065

^a^*p*< .05 compared to 10-HCPT injection group.

It can be seen from Table 1 that the half-life of free 10-HCPT was only 10 min, which was basically consistent with the literature report. Shorter drug half-lives are extremely detrimental to the efficacy, especially for the treatment of tumors. On the one hand, rapid clearance can seriously reduce the accumulation of chemotherapeutic drugs in tumor tissues and cells, which in turn affects the efficacy. On the other hand, the lack of tissue distribution selectivity directly leads to the occurrence of toxic side effects, which in turn affects patient tolerance. Therefore, how to prolong the half-life of 10-HCPT is the key to improve its efficacy and expand its clinical application. By contrast, the half-life of HES-10-HCPT-SS-Ly micelles is up to 4.58 hours, which is extremely beneficial for the accumulation of 10-HCPT in liver cancer tissues. The main reason why the polymer–drug conjugated micelles are selected as the delivery system in this study is that the properties of the polymer carrier can significantly affect the physical and chemical properties of the drug. HES, as a plasma substitute, has a high Mw and degree of substitution, showing excellent long-circulation effects *in vivo*. In addition, the bonds between HES and 10-HCPT has significant pH sensitivity and is stable under physiological conditions, resulting in a significant increase in the half-life of 10-HCPT. Meanwhile, the bioavailability of HES-10-HCPT-SS-Ly micelles is 50 times higher than that of 10-HCPT injection. It can be concluded that HES as a new generation of polymer carrier can not only improve the solubility of poorly soluble drugs, but also significantly improve the *in vivo* behavior of drugs, which also provides more theoretical support for subsequent pharmacodynamics and tissue distribution studies.

### 3.6. The pharmacodynamics of HES-10-HCPT-SS-Ly micelles

**Figure 6.**
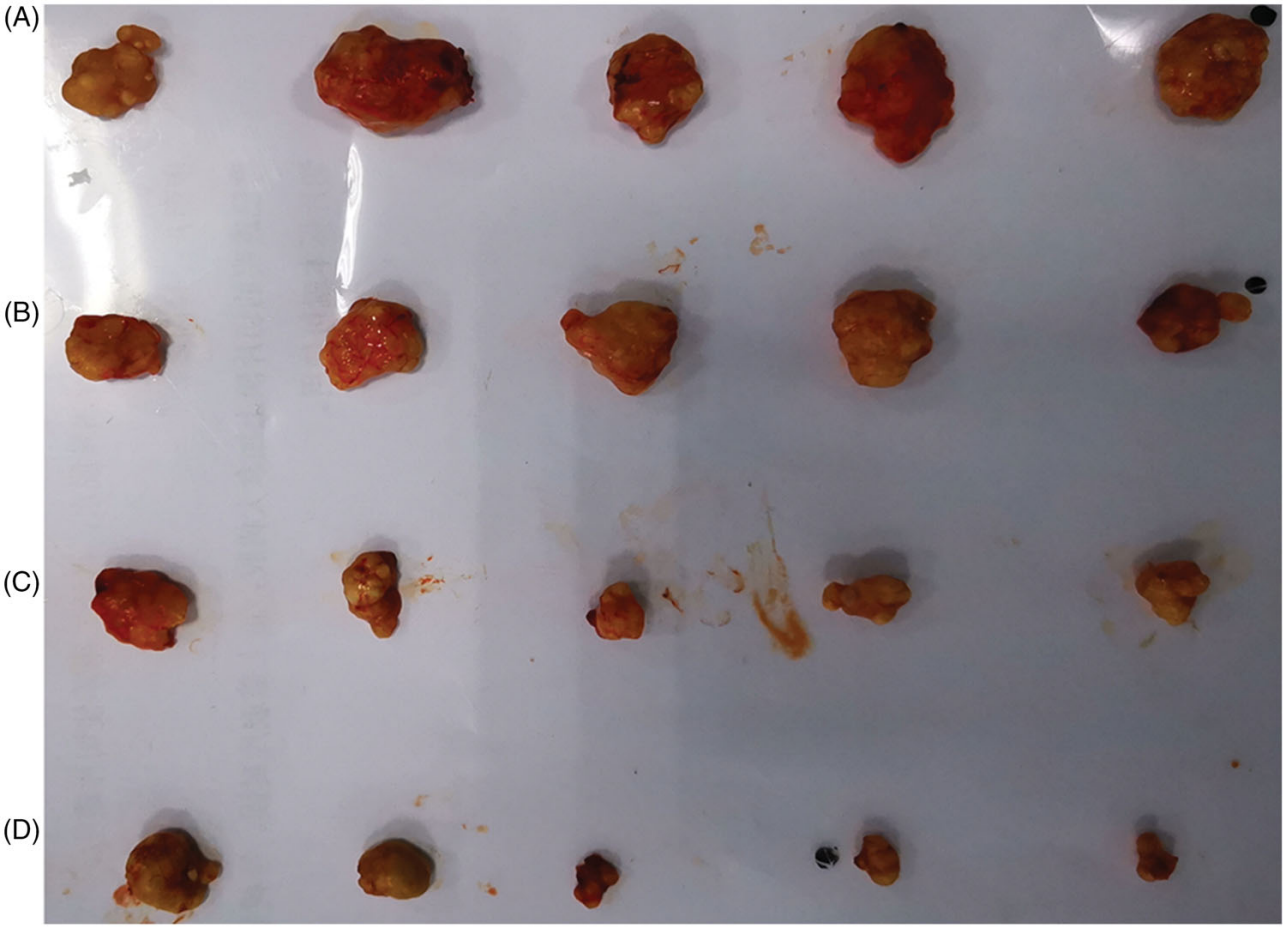
The excised tumor photo of HES-10-HCPT-SS-Ly micelle on node mice (five animals in each group) xenografted with Hep-G2 tumors. Group A: saline group; group B: 10-HCPT injection group; group C: non-targeted micelle group; group D: targeted micelle group.

The results of *in vivo* antitumor activity of HES-10-HCPT-SS-Ly micelles are shown in Table 2 and Figure 6.

**Table 2.** Anti-tumor effects of 10-HCPT preparations against Hep-G2 cell in nude mouse.

	Dose (mg/kg)	Body weight (g)		Tumor weight (g)	IRT (%)
		Initial	Final		
Normal saline	–	25.6	36.7	1.08 ± 0.13	–
10-HCPT injection	2	24.2	34.6	0.81 ± 0.11	26.2
Non-targeted micelle	2	24.5	35.8	0.23 ± 0.09	79.2^a^
Targeted micelle	2	23.6	35.7	0.11 ± 0.03	90.3^a^^,^^b^

a *p*< .05, compared with 10-HCPT injection group.

b *p*< .05, compared with non-targeted micelle group.

First, there is a significant difference in tumor volume among groups. The tumor volume of negative control group was much larger than that of other groups. The average tumor weight of the negative group, 10-HCPT injection group, HES-10-HCPT micelles, and HES-10-HCPT-SS-Ly micelles was 1.085 ± 0.132 g, 0.801 ± 0.106 g, 0.226 ± 0.087 g, 0.105 ± 0.034 g, respectively. It can be seen that 10-HCPT preparations can effectively inhibit the progression of the tumor to some extent, especially for the micelles group. This also shows that the efficacy of 10-HCPT can be fully utilized with the improvement of its physical and chemical properties. Compared with HES-10-HCPT micelles, HES-10-HCPT-SS-Ly micelles exhibited better antitumor activity. This may be due to the fact that more 10-HCPT enters the tumor cells under the mediation of Ly in the HES-10-HCPT-SS-Ly micelles group according to the results of the above MTT assay. Second, there are also significant differences in vascular irritation and tolerance. A number of irritating reactions were found in 10-HCPT injection, including vascular ulceration, violent reaction during injection, weight loss, and loss of locomotor activity, which resulted in a significant decrease in the tolerance of mice to 10-HCPT injection. This may be due to toxicity of 10-HCPT in the form of carboxyl groups. In comparison, HES-10-HCPT micelles and HES-10-HCPT-SS-Ly micelles group showed no important irritation. Meanwhile, the nude mice remained in a good state throughout the experiment. It can be inferred that the free 10-HCPT was significantly reduced in the blood circulation after the formation of HES-10-HCPT/HES-10-HCPT-Ly micelles, which in turn reduces systemic toxicity and irritation. Finally, HES-10-HCPT-Ly micelles showed better inhibition of tumor growth than HES-10-HCPT micelles group, and the corresponding tumor inhibition rate of each group was 90.3% and 79.2%. This may benefit from the mediation of Ly. It is well known that 10-HCPT is a class of drugs whose target is located in the nucleus (inhibiting the DNA topoisomerase I of the tumors), hence it must enter the cell to play the role of killing tumor cells. HES-10-HCPT-Ly micelles-loaded 10-HCPT can efficiently enter tumor cells through the mediation of Ly, which greatly improve the uptake efficiency of 10-HCPT. Thus, HES-10-HCPT-SS-Ly micelles showed satisfactory anti-tumor effect and low toxicity.

### 3.7. Tissue distribution of HES-10-HCPT-SS-Ly micelles

**Figure 7.**
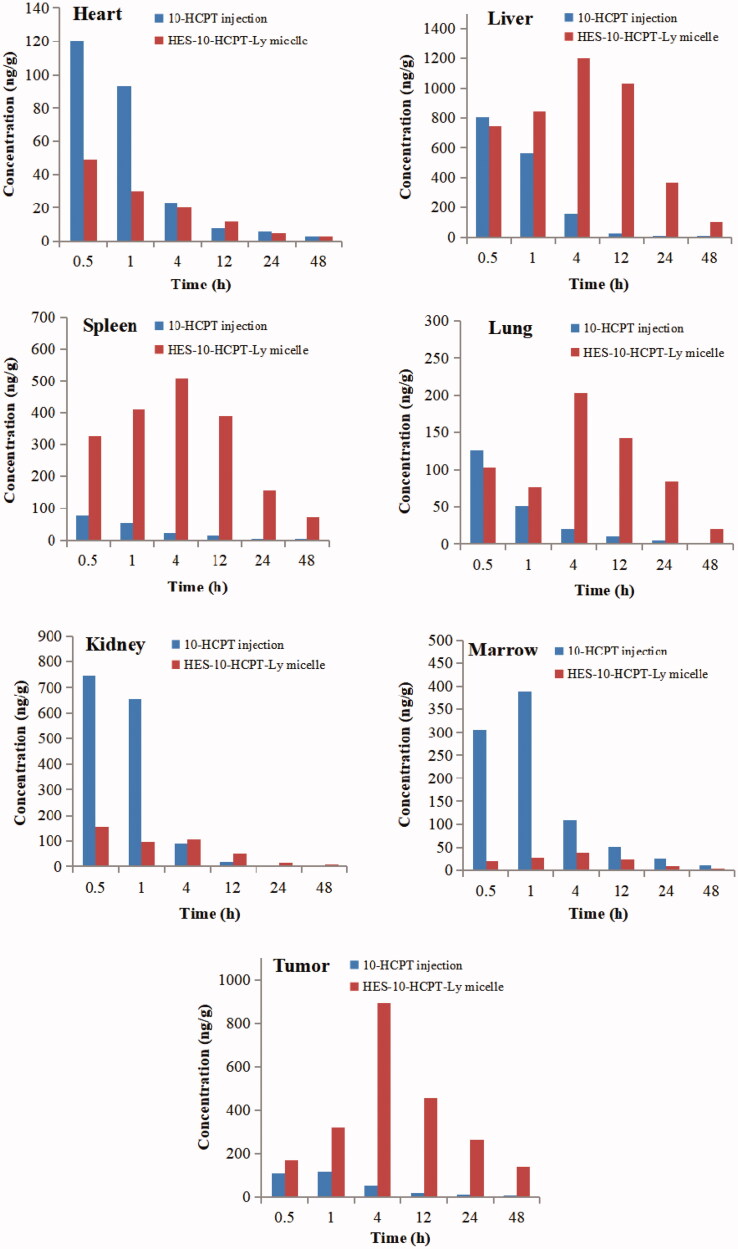
Tissue distribution profiles of 10-HCPT after intravenous 10-HCPT injection and HES-10-HCPT-Ly micelles administration of 1.5 mg/kg.

Tissue concentrations of 10-HCPT injection and HES-10-HCPT-SS-Ly micelles were evaluated in this paper, and the results are shown in Figure 7 and Table 3.

**Table 3.** Comparison of tissue of AUCs of 10-HCPT after i.v. administration of 10-HCPT injection and micelle at a dose of 1.5 mg/kg.

Tissues	AUC (ng/mL)	
	10-HCPT injection	HES-10-HCPT-Ly micelle
Heart	200.3 ± 21.5	157.6 ± 14.6
Liver	1354.2 ± 132.8	31673.9 ± 1983.5
Spleen	211.8 ± 24.1	6745.3 ± 352.7
Lung	367.9 ± 36.3	2467.2 ± 137.4
Kidney	1562.9 ± 148.8	1358.4 ± 127.1
Tumor	593.2 ± 46.8	16543.7 ± 975.6
Marrow	1956.3 ± 169.4	368.4 ± 40.8

It can be seen that the injection group showed a relatively higher drug concentration in the heart and kidney than HES-10-HCPT-SS-Ly micelles group, especially in the early stages. However, no significant difference was found in the AUC values of the two groups in the heart and kidney. In terms of liver, spleen, and lung, two formulations show significant differences. Compared with 10-HCPT injection group, HES-10-HCPT-SS-Ly micelles group exhibited higher drug concentrations in liver, spleen, and lung, and the AUC value reached 23-fold, 32-fold, and 7-fold, respectively. This may be related to the presence of a large number of macrophages in the above tissues, including Kupffer cell, alveolar macrophage. It is well known that nanoparticles have natural targeting to these macrophages, resulting in accumulation of nanoparticles in these tissues easily while HES-10-HCPT-SS-Ly micelles have the properties of nanoparticles and easily taken up by macrophages of these tissues. However, 10-HCPT injection belongs to small molecule solution, which can avoid the phagocytosis by RES system and quickly distribute to each organization. Thus, HES-10-HCPT-SS-Ly micelles have a higher concentration in these tissues. However, it was found that the uptake efficiency of micelles is much lower than HES-10-HCPT conjugate in terms of spleen and lung compared with our previous experimental results. On the one hand, the prepared micelles have better function of avoiding macrophage recognition than HES-10-HCPT conjugate. On the other hand, the content of macrophages in the lungs and spleen is much lower than that in the liver. Thus, the accumulation of HES-10-HCPT-SS-Ly micelles in the spleen and lungs is significantly reduced. What surprised us is that the prepared micelles can significantly increase the bioavailability and drug accumulation in the tumor tissue. This may be due to the fact that the half-life of 10-HCPT is significantly extended after being made into micelles. Excellent long-circulating properties allow micelles to fully utilize the EPR effects of tumor blood vessels to accumulate in tumor tissue, thereby significantly increasing the drug concentration of the tumor tissue. It is well known that the EPR effect is ineffective for small molecule solutions, hence 10-HCPT injection has no selectivity for tumor tissue accumulation. 10-HCPT concentration in the bone marrow significantly decreased with the preparation of HES-10-HCPT-SS-Ly micelles, and the AUC value of micelles group was sixfold lower than that of 10-HCPT injection group. As we know, 10-HCPT could induce the death of normal cell, especially for metabolically active cell, such as bone marrow stem cells, which resulted from *in vivo* distribution of 10-HCPT that lacked sufficient selectivity. However, HES-10-HCPT-SS-Ly micelles could not be taken up by marrow system due to the large particle size. Thus, the marrow toxicity of 10-HCPT significantly decreased with the preparation of micelles, which had important implications for the treatment of clinical disease.

## 4. Conclusions

HES-10-HCPT-SS-Ly micelles were prepared and characterized successfully. The micelles showed ideal particle size and pH/α-mylase/reduction responsive drug release. Meanwhile, HES-10-HCPT-SS-Ly micelles can obviously change *in vivo* behavior of 10-HCPT. In addition, HES-10-HCPT-SS-Ly micelles exhibited greater cytotoxicity on Hep-G2 cells and better *in vivo* antitumor activity in nude model bearing Hep-G2 cell because of the active targeting of Ly compared with HES-10-HCPT micelles and 10-HCPT injection.
